# Enhanced recombinant protein production and differential expression of molecular chaperones in *sf*-caspase-1-repressed stable cells after baculovirus infection

**DOI:** 10.1186/1472-6750-12-83

**Published:** 2012-11-07

**Authors:** Yiu-Kay Lai, John T-A Hsu, Chih-Chieh Chu, Teng-Yuan Chang, Kao-Lu Pan, Chih-Chien Lin

**Affiliations:** 1 Institute of Biotechnology, Department of Life Science, National Tsing Hua University, No. 101, Section 2, Kuang-Fu Road, Hsinchu, 30013, Taiwan, R.O.C; 2 Division of Biotechnology and Pharmaceutical Research, National Health Research Institutes, 35 Keyan Road, Zhunan, Miaoli, 35053, Taiwan, R.O.C; 3 Department of Cosmetic Science, Providence University, 200 Chung-Chi Road, Shalu, Taichung, 43301, Taiwan, R.O.C

**Keywords:** Apoptosis, Baculovirus, Chaperone, RNA interference, *Sf*-caspase-1

## Abstract

**Background:**

There are few studies that have examined the potential of RNA inference (RNAi) to increase protein production in the baculovirus expression vector system (BEVS). *Spodoptera frugiperda* (fall armyworm) (*Sf*)-caspase-1-repressed stable cells exhibit resistance to apoptosis and enhancement of recombinant protein production. However, the mechanism of recombinant protein augmentation in baculovirus-infected Caspase-repressed insect cells has not been elucidated.

**Results:**

In the current study, we utilized RNAi-mediated *Sf*-caspase-1-repressed stable cells to clarify how the resistance to apoptosis can enhance both intracellular (firefly luciferase) and extracellular (secreted alkaline phosphatase [SEAP]) recombinant protein production in BEVS. Since the expression of molecular chaperones is strongly associated with the maximal production of exogenous proteins in BEVS, the differential expression of molecular chaperones in baculovirus-infected stable cells was also analyzed in this study.

**Conclusion:**

The data indicated that the retention of expression of molecular chaperones in baculovirus-infected *Sf*-caspase-1-repressed stable cells give the higher recombinant protein accumulation.

## Background

Programmed cell death, also known as apoptosis, is a normal physiological cell suicide program that is highly conserved among vertebrates and invertebrates [1,2]. Apoptotic cells undergo a series of dramatic and characteristic alterations in cellular morphology, such as DNA fragmentation, chromatin condensation, cytoskeleton reorganization, and plasma membrane blebbing [3]. Apoptosis plays an important role during development and tissue homeostasis eliminating discarded cells from the organism, including damaged and virus-infected cells. For this reason, apoptosis acts as a host protection mechanism by which virus-infected cells are removed to limit the proliferation of viruses [4,5]. Thus, to overcome this defense response in cells, baculoviruses carry anti-apoptotic genes to inhibit programmed cell death [6-8].

**Figure 1:**
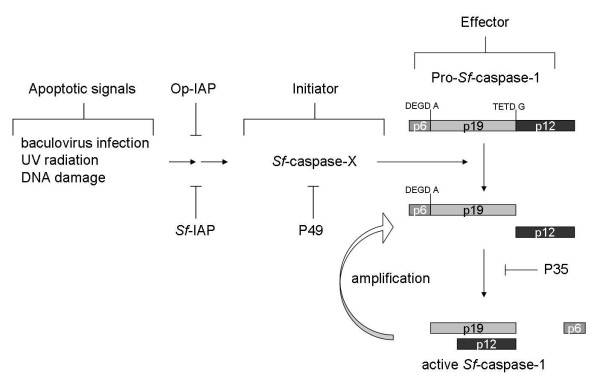
**The apoptosis pathway in *S. frugiperda *cells.** Apoptotic signals trigger the activation of initiator *Sf*-caspase-X in *S. frugiperda* cells. The Op-IAP and *Sf*-IAP suppress the activation of *Sf*-caspase-X, which proteolytically activates downstream effector caspases, including *Sf*-caspase-1, by cleavage at the large-small subunit junction. The activated effector caspases can amplify this processing and different steps are inhibited by P49 and P35.

*Spodoptera frugiperda* caspase-1 (*Sf*-caspase-1), the most studied effector caspase of Lepidoptera, is the principal effector caspase of *S. frugiperda* 9 (Sf9) cells, and is activated by various death stimuli, including baculovirus infection, ultraviolet (UV) irradiation, and over-expression of pro-apoptotic genes (Figure 1) [8-10]. Two types of anti-apoptotic genes have been identified in baculoviruses, *p35* (p49) and inhibitor of apoptosis (IAP) [6-8]. The *p35* gene from *Autographa californica* multicapsid nucleopolyhedrovirus (AcMNPV) and *p49* gene from *Spodoptera littoralis* nucleopolyhedrovirus (SlNPV) are required to prevent apoptosis during the virus infection of *S. frugiperda* cells, such as Sf9 and Sf21 [2,11,12]. The Op-IAP from the *Orgyia pseudotsugata* multicapsid nucleopolyhedrovirus (OpMNPV) and *Sf*-IAP from the host, *S. frugiperda* cells, also suppress the apoptosis process (Figure 1) [1,2].

The baculovirus expression vector system (BEVS) is a powerful tool used for the expression of foreign proteins in numerous insect cells, including Sf9, Sf21 cells and *Trichoplusi ni* cells [13-15]. Therefore, recombinant protein production using a BEVS can be used for many pharmaceutical applications [16-18].

Exposure of cells to stressors, including high temperatures or a wide variety of physical and chemical insults, induces the expression of heat-shock proteins (HSPs) in cells [19,20]. HSPs are molecular chaperones responsible for maintaining cell homeostasis and promoting cell survival [21]. Baculovirus infection also serves as a stress factor that can activate both death-inducing and cellular-protective pathways, and the heat-shock response is important for baculovirus replication in insect cells [22]. Moreover, the rate-limited expression of endoplasmic reticular (ER) molecular chaperones is strongly associated with the maximal expression of exogenous proteins by BEVS [23].

Few studies have examined the potential of RNA inference (RNAi) to increase protein production in the BEVS [24,25], however, several studies have demonstrated the efficiency of this approach in both insect cells and larvae [26,27]. In our previous studies, we used DNA vector-based approaches with endogenously expressed double-stranded RNA (dsRNA) to silence its target gene, *Sf*-caspase-1, in Sf9 cells. Therefore, the *Sf*-caspase-1 mRNA level and plasmid copy number in the *Sf*-caspase-1-repressed stable cells were examined [25]. In addition, *Sf*-caspase-1-repressed stable cells exhibited resistance to apoptosis and enhancement of recombinant protein production [25,28]. These results were consistent with later findings in *T. ni* cells [24]; however, the mechanism of recombinant protein augmentation in baculovirus-infected Caspase-repressed insect cells was not determined. Therefore, in this present study, we use RNAi-mediated *Sf*-caspase-1-repressed stable cells to clarify how resistance to apoptosis could enhance both intracellular (firefly luciferase) and extracellular (secreted alkaline phosphatase [SEAP]) recombination protein production by BEVS. Furthermore, the differential expression of molecular chaperones in baculovirus-infected stable cells was also analyzed in this study.

## Results

### Stable transfection plasmid verification by genomic DNA PCR and RT-PCR in Sf9/pIBdsCasp-1 and Sf9/pIBdsCasp-2 cells

**Figure 2:**
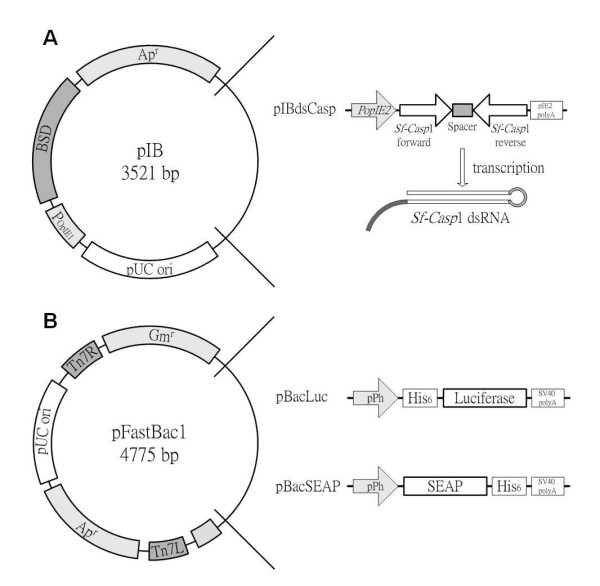
**Inverted repeat vectors for the endogenous expression of *Sf-casp*1 dsRNA (A) and the recombinant baculovirus vectors (B).** The resulting stably transfected cell lines carry a plasmid that contains an 800 bp stretch of DNA sequence of *Sf-casp*1 mRNA in both forward and reverse orientations, containing a spacer of 100 bp between each element. Recombinant baculovirus vectors, pBacLuc and pBacSEAP contained gene fragments encoding the luciferase and SEAP, respectively. Both genes were expressed under the control of the pPh promoter.

**Figure 3:**
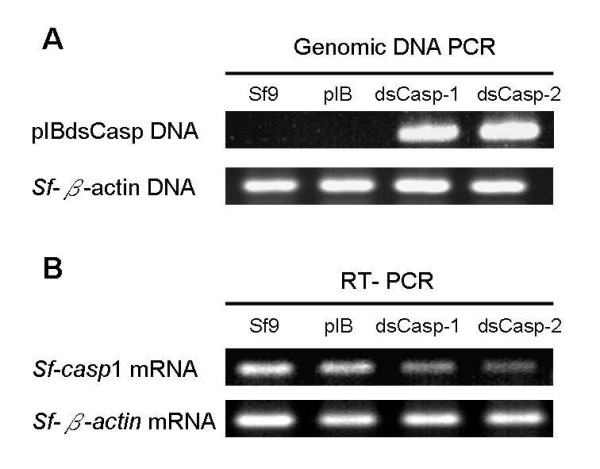
**Genomic DNA PCR (A) and RT-PCR (B) analysis in Sf9/pIBdsCasp-1 and Sf9/pIBdsCasp-2 cells.** Genomic DNA and total RNA of the cells (Sf9, Sf9/pIB, Sf9/pIBdsCasp-1 and Sf9/pIBdsCasp-2) were extracted and analyzed by PCR and RT-PCR, respectively. The corresponding specific primer sequences and PCR cycles are presented in Table 1. pIB, Sf9/pIB cells; dsCasp-1, Sf9/pIBdsCasp-1 cells; dsCasp-2, Sf9/pIBdsCasp-2 cells.

Stable cells (Sf9/pIB, Sf9/pIBdsCasp-1, and Sf9/pIBdsCasp-2 cells) were established by transfection of the control vector pIB or vector pIBdsCasp (Figure 2), and subsequently selected by BSD [25]. After more than 20 passages, stable cell lines were analyzed by genomic DNA PCR and RT-PCR to examine the inverted-repeat DNA sequence of *Sf*-caspase-1 and endogenous expressed *Sf*-caspase-1 mRNA quantity. The corresponding primers and PCR cycle number used for the experiments in Figure 3 are shown in Table 1. As shown for the genomic DNA PCR performed in Figure 3A, we supposed the pIBdsCasp vectors were stably integrated with Sf9 cellular genomic DNA. In addition, the data also showed that levels of *Sf*-casp1 mRNA in Sf9/pIBdsCasp-1 and Sf9/pIBdsCasp-2 cells were apparently lower than those observed in Sf9 and Sf9/pIB cells (Figure 3B). Furthermore, control actin mRNA was not obviously affected in either of the cell lines. These results demonstrated that *Sf*-caspase-1 mRNA was successfully suppressed by *Sf*-caspase-1 dsRNA in both Sf9/pIBdsCasp-1 and Sf9/pIBdsCasp-2 cells. In our previous study, we tested the expression of P35, a substrate inhibitor of *Sf*-caspase-1, in Sf9/pIBdsCasp-1 and Sf9/pIBdsCasp-2 cells, and found that the level of P35 in pIBdsCasp transfected cells was evidently higher than that found in normal cells [25]. Thus, we suggested that *Sf*-caspase-1 is suppressed in these *Sf*-caspase-1-repressed cells.

**Table 1:** Primers used in PCR and RT-PCR analyses

**Gene**	**Protein/product**	**Predicted location**	**Description**	**Primer sequence**	**PCR cycle**	**Reference**
*Sf-casp*1	*Sf-casp*1 dsRNA	nucleus/cytosol	pIBdsCasp DNA, forward	5^′^-GATATGGAGAAGATCTGGCAC-3^′^	35	[25]
				5^′^-GTGGGGCAGGGCGTAGCC-3^′^		
			pIBdsCasp DNA, reverse			
*Sf-β-actin*	β-Actin	cytosol	*Sf-β-actin* DNA/mRNA, forward	5^′^-CAATCTGTCACCTTGGCA-3^′^	25	[25]
				5^′^-GACAATACAAACTAAGATTTA-3^′^		
			*Sf-β-actin* DNA/mRNA, reverse			
*Sf-casp*1	*Sf*-Caspase-1	cytosol	*Sf-casp*1 mRNA, forward	5^′^-TGTCAAACACCTTTTATG-3^′^	25	[25]
				5^′^-TATTATGACACATGGGCA-3^′^		
			*Sf-casp*1 mRNA, reverse			
*atx1*	Copper chaperone	cytosol	*atx1* mRNA, forward	5^′^-ATGTCATCTACACACATTTT-3^′^	25	[29]
			*atx1* mRNA, reverse			
				5^′^-CTATTGTGTGCCAACATAAG-3^′^		
*cypA*	Cyclophilin A (CypA)	cytosol	*cypA* mRNA, forward	5^′^-ATGGCTTTACCCCGAGTTT-3^′^	25	[29]
				5^′^-TGGTTTGTGAAGTCACCTCCT-3^′^		
			*cypA* mRNA, reverse			
*cypB*	Cyclophilin B (CypB)	secreted/ER	*cypB* mRNA, forward	5^′^-GCGCTTTCGCAGCAGCTCTT-3^′^	25	[29]
				5^′^-AGCCCTGGCCTTCTGGTTT-3^′^		
			*cypB* mRNA, reverse			
*hsc70*	Heat shock cognate	ER	*hsc70* mRNA, forward	5^′^-ATGATCAAAATGCGGTGGA-3^′^	25	[30]
	70 (GRP78-like)			5^′^-AGCCACATATGAGGGAGTGAT-3^′^		
			*hsc70* mRNA, reverse			
*hsp90*	Heat shock protein	ER	*hsp90* mRNA, forward	5^′^-ATGCCCGAAGAAATGCAG-3^′^	25	[31]
	90 (HSP90)					
			*hsp90* mRNA, reverse			
				5^′^-TGTACAGCTCCTTGCCGCT-3^′^		
*calr*	Calreticulin (CRT)	cytosol	*calr* mRNA, forward	5^′^-ATTCTATTCGTGGCCAGTCC-3^′^	25	[29]
				5^′^-GAACTTTCCAGCAGTCAGCTT-3^′^		
			*calr* mRNA, reverse			
*pdi*	Protein disulfide	ER	*pdi* mRNA, forward	5^′^-GCCATTGACGCTGACGAA-3^′^	25	[29]
	isomerase (PDI)		*pdi* mRNA, reverse	5^′^-CCTCGCTCAACAAGTGCTGT-3^′^		

ER, endoplasmic reticulum.

### Accumulated SEAP expression by Sf9/pIBdsCasp-1 and Sf9/pIBdsCasp-2 cells

**Figure 4:**
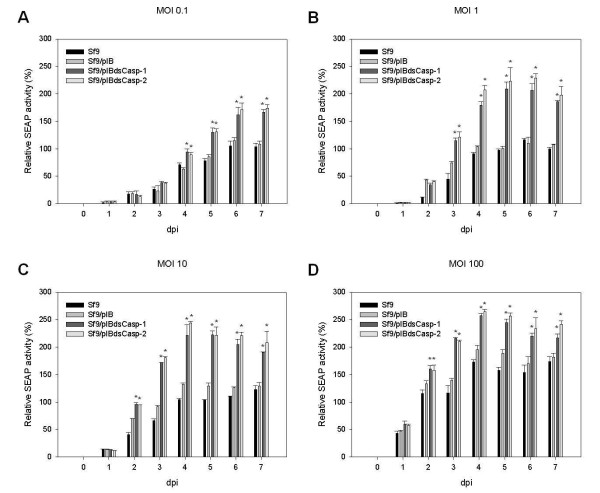
**Accumulated SEAP expression of Sf9/pIBdsCasp-1 and Sf9/pIBdsCasp-2 cells.** Cells (Sf9, Sf9/pIB, Sf9/pIBdsCasp-1 and Sf9/pIBdsCasp-2) were infected with rBacSEAP at MOIs of 0.1 (**A**), 1 (**B**), 10 (**C**) and 100 (**D**). At each day post-infection, culture media were analyzed for relative specific SEAP activity. Data is presented as the mean ± S.D. of two independent experiments, and statistically analyzed using the Student’s *t*-test. *p < 0.05, compared to corresponding controls.

SEAP is a secreted (extracellular) recombinant protein that can be assayed in culture medium using a standard activity assay. Thus, we used rBacSEAP to infect the Sf9/pIBdsCasp-1 and Sf9/pIBdsCasp-2 cells at a multiplicity of infection (MOI) of 0.1-100 and analyzed the accumulated SEAP expression in the culture medium every day post infection (dpi). At 2–4 dpi, the accumulated SEAP expression in rBacSEAP-infected Sf9/pIBdsCasp-1 and Sf9/pIBdsCasp-2 cells was obviously higher than that in the control group, Sf9 and Sf9/pIB cells, at all MOIs (Figure 4). Moreover, at an MOI of 1 and 10, accumulated SEAP expression in rBacSEAP-infected Sf9/pIBdsCasp-1 and Sf9/pIBdsCasp-2 cells was approximately two-fold higher than that in the control group after 4 dpi (Figure 4B and 4C). Moreover, the difference of accumulated SEAP expression between normal and *Sf*-caspase-1-repressed stable cells increased with MOI, such as that at MOIs of 10 and 100 (Figure 4). These data demonstrated that the secreted recombinant protein production in *Sf*-caspase-1-repressed stable cells was higher than that in normal insect cell after baculovirus infection.

### Luciferase expression in Sf9/pIBdsCasp-1 and Sf9/pIBdsCasp-2 cells

**Figure 5:**
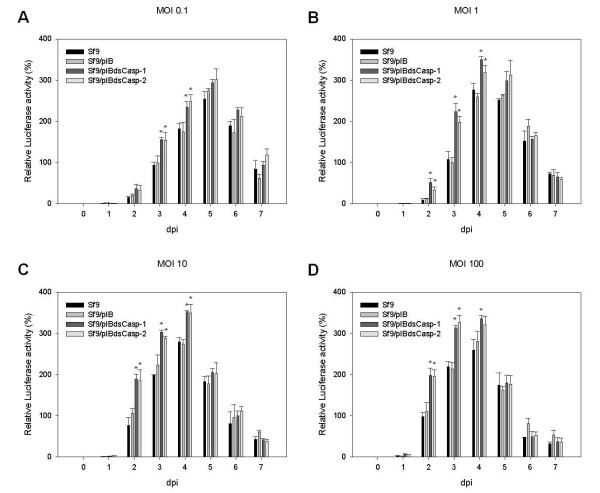
**Luciferase expression in Sf9/pIBdsCasp-1 and Sf9/pIBdsCasp-2 cells.** Cells (Sf9, Sf9/pIB, Sf9/pIBdsCasp-1 and Sf9/pIBdsCasp-2) were infected with rBacLuc at MOIs of 0.1 (**A**), 1 (**B**), 10 (**C**) and 100 (**D**). At each day post-infection, cells were collected and analyzed for relative specific luciferase activity. Data is presented as the mean ± S.D. of two independent experiments, and statistically analyzed using the Student’s *t*-test. *p < 0.05, compared to corresponding controls.

To estimate different kinds of recombinant protein production in *Sf*-caspase-1-repressed stable cells, we use an intracellular recombinant protein, luciferase, as the assay target. The rBacLuc-infected Sf9/pIBdsCasp-1 and Sf9/pIBdsCasp-2 cells showed higher luciferase activities than that in control cells at all MOIs at 2 or 3 dpi (Figure 5). However, at all MOIs, the difference of luciferase expression was not apparent at >5 dpi (Figure 5) and might result from the lysis of virus-infected cells during the later phase of infection releasing intracellular proteins [32].

### SEAP expression at each day’s period in Sf9/pIBdsCasp-1 and Sf9/pIBdsCasp-2 cells

**Figure 6:**
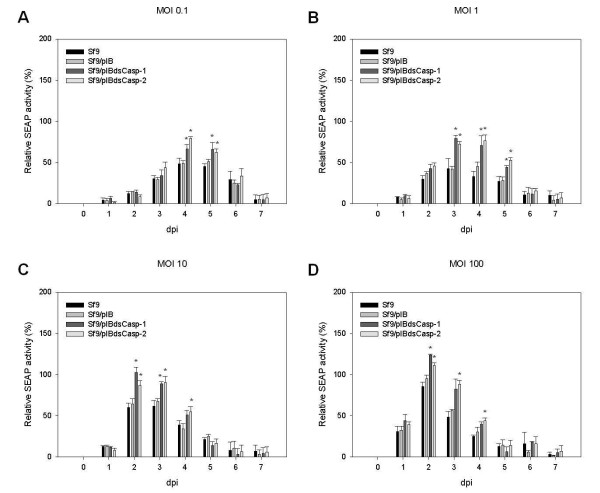
**SEAP expression at each day’s period in Sf9/pIBdsCasp-1 and Sf9/pIBdsCasp-2 cells.** Cells (Sf9, Sf9/pIB, Sf9/pIBdsCasp-1 and Sf9/pIBdsCasp-2) were infected with rBacSEAP at MOIs of 0.1 (**A**), 1 (**B**), 10 (**C**) and 100 (**D**). At each day post-infection, old culture media were replaced with fresh media and analyzed for relative specific SEAP activity. Data is presented as the mean ± S.D. of two independent experiments, and statistically compiled by Student’s *t*-test. *p < 0.05, compared to corresponding controls.

To further investigate the differences of recombinant protein production between the normal and *Sf*-caspase-1-repressed stable cells, specific SEAP activity at every 24 hour period of time in the culture media of rBacSEAP infected cells was analyzed. At an MOI of 0.1 and 1, SEAP activities in culture medium of Sf9/pIBdsCasp-1 and Sf9/pIBdsCasp-2 cells were higher than that of normal cells at 3–5 dpi (Figure 6A and 6B). Moreover, at an MOI of 10 and 100, peak SEAP expression in *Sf*-caspase-1-repressed and normal cells occurred at 2–4 dpi (Figure 6C and 6D). Although during the assay the replacement of culture medium of virus-infected cells may affect not only the energy sources for protein expression but also virus replication in cells, the difference of recombinant protein production between *Sf*-caspase-1-repressed and normal cells may be caused by the diverse cellular states that occur during the middle phase of the infection process.

### Expression of molecular chaperones in baculovirus-infected Sf-caspase-1-repressed stable cells

**Figure 7:**
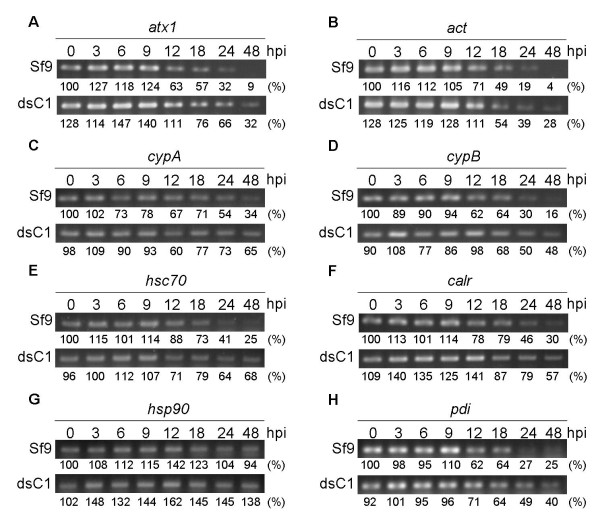
**RT-PCR analysis of molecular chaperones in baculovirus-infected *Sf*-caspase-1-repressed stable cells.** Total mRNA of baculovirus-infected cells, Sf9 and Sf9/pIBdsCasp-1, at each hour post infection (hpi) were extracted and analyzed by RT-PCR. The marked numbers below images were relative mRNA level (%), which were normalized with rRNA and compared with corresponding mRNA level of Sf9 cell at 0 hpi. The corresponding specific primer sequences and PCR cycles were presented in Table 1. dsC1, Sf9/pIBdsCasp-1 cells; *act*, β-actin.

The heat shock response is important for baculovirus replication in insect cells and the expression of ER molecular chaperones is strongly associated with the maximal expression of exogenous proteins in BEVS [22,23]. Therefore, we analyzed the mRNA expression of some molecular chaperones involved in virus replication, including copper chaperone, cyclophilins, HSPs, calreticulin (CRT), and protein disulfide isomerase (PDI). The β-actin mRNA level in Sf9 and Sf9/pIBdsCasp-1 cells was relatively equivalent at each time point analyzed and decreased at 9–48 hours post-infection (hpi) (Figure 7B). However, the mRNA expression of chaperones, including *atx1*, *cypA*, *cypB*, *hsc70*, *calr*, and *pdi*, exhibited differential expression between baculovirus-infected Sf9 and Sf9/pIBdsCasp-1 cells at 9–48 hpi (Figure 7). Most chaperones demonstrated an induction response for virus infection within 9 hpi. Furthermore, expression of *hsp90* was persistent during the infection process and no dramatic difference was observed between normal and *Sf*-caspase-1-repressed cells (Figure 7G). In contrast, expression of *hsc70* did not show the same stable expression (Figure 7E). These data are consistent with a study performed by Nobiron et al. [33] that identified the cause of this effect to be HSC70, a virus-induced member of the HSP70 family.

## Discussion

In this study, our data demonstrated that both intracellular (luciferase) and extracellular (SEAP) recombinant protein production in *Sf*-caspase-1-repressed stable cells was higher than that in normal insect cell after baculovirus infection (Figure 4 and 5). Although luciferase expression did not correlate with accumulated SEAP expression, it was apparent that both extracellular and intracellular recombinant protein production in *Sf*-caspase-1-repressed stable cells was higher than for parental cells (Figures 4 and 5). Thus, these data indicate that *Sf*-caspase-1-repressed stable cells express a higher amount of recombinant protein in BEVS, consistent with previous studies in *S. frugiperda* cells by our group [25] and in *T. ni* cells by Bentley’s group [24,34]. Hence, we can suggest that the apoptotic repressed insect cells have greater recombinant protein production when infected with recombinant baculovirus, providing an effective production tool for BEVS. Besides, results also indicated that the difference of recombinant protein production between *Sf*-caspase-1-repressed and normal cells may be caused by the diverse cellular states that occur during the middle phase of the infection process (Figure 6).

Molecular chaperones are important and highly associated with the state of cells. The results of molecular chaperone mRNA levels in baculovirus-infected normal and *Sf*-caspase-1-repressed stable cells demonstrated that these cells were appeared in different states during the infection progression (Figure 7). Therefore, we assumed that this is a key explanation for baculovirus-infected *Sf*-caspase-1-repressed stable cells have a higher recombinant protein production than that in normal cells. The persistent expression of molecular chaperones in baculovirus-infected *Sf*-caspase-1-repressed stable cells resulted in a higher recombinant protein production than that in normal cells, which can be suggested by some earlier studies that focused on the co-expression of molecular chaperones, Bip (GRP78), Calreticulin and Calnexin in BEVS to improve the recombinant production and secretion from cells and larvas [35-38]. Therefore, we also suppose that expression of molecular chaperon in combined with RNAi technique may have opportunity to further improve BEVS.

During the baculovirus infection process, the condition of infected insect cells often represents key factors that affect both baculovirus replication and recombinant production. Therefore, there appears to be a distinct difference of the baculovirus infection process that occurs in normal and *Sf*-caspase-1-repressed cells. We proposed that the *Sf*-caspase-1-repressed stable cells have a different status and this response improves the ability of infected cells to express a higher amount of recombinant proteins.

## Conclusions

In summary, the differential expression of molecular chaperones in baculovirus-infected *Sf*-caspase-1-repressed stable cells affects the production of recombinant protein when compare with normal cells. Therefore, the current study identified critical virus-cell interactions that are likely to improve the development of BEVS in future studies.

## Methods

### Construction of plasmids and recombinant baculovirus

Stable transfection plasmids were constructed using the pIB vector (Invitrogen, Carlsbad, CA) as the backbone. The 5’ end 800 base pairs (bp) forward and reverse DNA fragments of the *Sf*-caspase-1 (*Sf-casp*1) gene were amplified by polymerase chain reaction (PCR) from the genomic DNA extracted from Sf9 cells. The resulting construct was named pIBdsCasp, containing an inverted-repeat sequence of *Sf-casp*1 under the control of the *opIE2* promoter, as described previously [25] and shown in Figure 2A. Illustrations of pBacLuc and pBacSEAP are provided in Figure 2B, and the construction of these plasmids was performed using the Bac-to-Bac system according to the manufacturer’s protocols (Invitrogen) as described previously [25,28]. Luciferase and SEAP were expressed in rBacLuc-, and rBacSEAP-infected insect cells, respectively. Recombinant baculovirus were amplified twice and titers were determined by the end-point dilution method [39].

### Cell culture and transfection

Sf9 cells were cultured in Grace’s insect cell culture medium (Invitrogen) with 10% (v/v) heat inactivated fetal bovine serum (FBS; Hyclone, Logan, UT) at 27°C. Cell density was determined by hemocytometer counts and cell viability was evaluated by the Trypan Blue exclusion method. The pIB vector or pIBdsCasp plasmids were transfected into Sf9 cells by Transfast reagent (Promega, Madison, WI) and stable cell lines were selected by the addition of 60 μg Blasticidin (BSD)/mL (Invitrogen), as previously described [25]. Stable clones encoding pIB vector and pIBdsCasp plasmid were designated Sf9/pIB and Sf9/pIBdsCasp (Sf9/pIBdsCasp-1 and Sf9/pIBdsCasp-2) cells, respectively.

### PCR and RT-PCR analysis

Primers used for PCR and reverse transcription PCR (RT-PCR) analyses are listed in Table 1. The genomic DNA was extracted from 5 × 10^4^ stable insect cells using the Dneasy Tissue Extraction kit (Qiagen, Hilden, Germany) and 500 ng of extracted genomic DNA was used in each 50 μL PCR. For genomic DNA PCR analysis, after an initial incubation at 94°C for 4 min, the reaction mixture was subjected to 25 or 35 cycles (Table 1) of PCR amplification at 94°C for 15 s, at 55°C for 30 s, and at 72°C for 1 min. PCR products were resolved by 1% agarose gel electrophoresis and analyzed after ethidium bromide staining.

Total RNA were isolated from cells using Trizol reagent (Invitrogen) according to standard protocols [40] and RNA concentrations were determined by UV absorption at 260 nm. To avoid DNA contamination, RNA samples were treated with RNase-free DNase I (Invitrogen) for 20 min at 37°C. For each RT-PCR, 200 ng of RNA were used in a 50 μL reaction. A reverse transcription step (50°C for 30 min and 94°C for 2 min) was followed by 25 cycles (Table 1) of denaturation at 94°C for 15 s, annealing at 55°C for 30 s, and extension at 72°C for 1 min. RT-PCR products were resolved by 1.2% agarose gel electrophoresis and analyzed after ethidium bromide staining. The ribosomal RNA (rRNA) of insect cells served as a loading control in the RT-PCR analysis.

### Luciferase and SEAP activity analyses

For luciferase activity analysis, enzyme activity in the cell lysate from 2 × 10^4^ cells was determined by the Luciferase reporter assay system (Promega) according to the manufacturer’s procedures and analyzed by a Victor^2^ Multilabel Counter (PerkinElmer Life Sciences, Norwalk, CT). Specific luciferase activities are presented as mean values normalized against those of the control (infected Sf9 cells).

For SEAP activity analysis, production of SEAP in 10 μL cultured medium was quantified by using a p-nitrophenylphosphate-based (pNPP) light absorbance time course method [41] and also analyzed by a Victor^2^ Multilabel Counter. The specific SEAP activities are presented as mean percentage relative to control (infected Sf9 cells).

### Statistical analysis

Quantitative data were analyzed using Student’s *t*-test and presented as means ± standard deviation (S.D.) of two or three independent experiments. The *P*-values <0.05 were considered significant.

## Competing interests

The authors declare that they have no competing interests.

## Authors’ contributions

YKL and JTAH designed the approach, helped with experimental design and data analysis, participated with manuscript preparation and editing. CCC, TYC and KLP helped with experiments execution, provided bioinformatics research and participated with manuscript preparation and editing. CCL participated with experiments execution and data interpretation, participated with prepared and submitted the manuscript. All authors read and approved the final manuscript.
